# Activation of OX40 and CD27 Costimulatory Signalling in Sheep through Recombinant Ovine Ligands

**DOI:** 10.3390/vaccines8020333

**Published:** 2020-06-22

**Authors:** José Manuel Rojas, Alí Alejo, Jose Miguel Avia, Daniel Rodríguez-Martín, Carolina Sánchez, Antonio Alcamí, Noemí Sevilla, Verónica Martín

**Affiliations:** 1 Centro de Investigación en Sanidad Animal (CISA-INIA), Instituto Nacional de Investigación y Tecnología Agraria y Alimentaria, Valdeolmos, 28130 Madrid, Spain; rojas.jose@inia.es (J.M.R.); alejo@inia.es (A.A.); Josemiguel.avia@fpclaudiogalenomadrid.es (J.M.A.); rodriguez.daniel@inia.es (D.R.-M.); sevilla@inia.es (N.S.); 2 Centro de Biología Molecular Severo Ochoa, Consejo Superior de Investigaciones Científicas and Universidad Autónoma de Madrid, Cantoblanco, 28049 Madrid, Spain; carolina_s@cbm.csic.es (C.S.); aalcami@cbm.csic.es (A.A.)

**Keywords:** T cell costimulatory signal, ovine immunology, TNF receptor, OX40, CD27

## Abstract

Members of the tumour necrosis factor (TNF) superfamily OX40L and CD70 and their receptors are costimulating signalling axes critical for adequate T cell activation in humans and mice but characterisation of these molecules in other species including ruminants is lacking. Here we cloned and expressed the predicted ovine orthologues of the receptors OX40 and CD27, as well as soluble recombinant forms of their potential ovine ligands, *Oa*OX40L and *Oa*CD70. Using biochemical and immunofluorescence analyses, we show that both signalling axes are functional in sheep. We show that oligomeric recombinant ligand constructs are able to induce signalling through their receptors on transfected cells. Recombinant defective human adenoviruses were constructed to express the soluble forms of *Oa*OX40L and *Oa*CD70. Both proteins were detected in the supernatant of adenovirus-infected cells and shown to activate NF-κB signalling pathway through their cognate receptor. These adenovirus-secreted *Oa*OX40L and *Oa*CD70 forms could also activate ovine T cell proliferation and enhance IFN-γ production in CD4^+^ and CD8^+^ T cells. Altogether, this study provides the first characterisation of the ovine costimulatory OX40L-OX40 and CD70-CD27 signalling axes, and indicates that their activation in vivo may be useful to enhance vaccination-induced immune responses in sheep and other ruminants.

## 1. Introduction

One of the major obstacles for generating safe and more effective vaccines that stimulate long lasting protective immunity is the lack of predictive immune correlates with protection. T cell-mediated responses against pathogens can result in potent and long-term protection against diseases, which can be critical to improve the outcome of vaccination. T cells not only mediate effector mechanisms such as cytotoxicity, but they also modulate immunity through helper mechanisms that adequately polarise the immune response towards the elimination of a pathogen. The tumour necrosis factor (TNF) and receptor (TNFR) superfamilies are fundamental in the co-stimulation of many types of immune reaction promoted by T cell help [1], like antibody secreting plasma cell activation or memory cell differentiation [2]. A number of costimulatory interactions in the TNF/TNFR superfamily, such as those between OX40L/OX40 or CD70/CD27, are implicated in controlling lymphocyte activation, proliferation and survival during viral immunity [3].

OX40 is predominantly expressed on activated T cells, in which its engagement by OX40L, its ligand typically expressed on conventional dendritic cells, expands effector T cells and promotes their survival and memory cell generation. OX40L ligation on OX40 signals through the NF-κB pathway as an independent signalling unit and it may also activate the phosphatyidl-inositol-3 kinase (PI3K) pathways when acting in concert with the T cell receptor [4]. One important role of this costimulatory pathway is the generation of CD4^+^ T cell responses including memory cell generation [5]. Collectively, OX40 is known to control the number of effector T cells that form during primary or secondary immune responses as well as the frequency of memory cells that are generated, reviewed in [4,6]. While directly affecting T cell expansion and survival, OX40 signalling has also been shown to indirectly affect cytokine production, thereby skewing the T response depending on the context. Moreover, OX40 signalling has also been found to be critical for CD8 T cell activation in several contexts including viral infections and immunopathology. OX40L is typically present on the surface of antigen-presenting cells as a 34 kDa glycosylated type II trans-membrane protein trimer, like other members of this family [7,8]. The extracellular C-terminal domain of OX40L is a structurally compact TNF homology domain (THD) that organises into a characteristic “jelly roll” beta-sandwich structure and assembles into distinctive open trimers that bind to its trimeric OX40 [9]. The receptor monomers bind the outside of the cork-shaped ligand at the interface between two monomers which constitute the basic signalling unit in this and most other TNF/TNFR interactions. Further receptor clustering at the cell membrane is also essential for efficient signalling [10].

Likewise, the interaction between CD70 and its receptor CD27 requires a trimeric CD70 complex interacting with three CD27 molecules on the cell membrane. The stimulation of CD27, a TNF superfamily receptor constitutively expressed on a large range of T-cells, NK-cells, and B-cells, through its ligand CD70 [11] activates the canonical and the alternative NF-κB pathways [12]. CD27 engagement on T cells is essential for memory cell differentiation while its engagement on B cell promotes plasma cell formation and enhances IgG production [13]. CD27 activation appears thus critical for the development of long-lasting adaptive immunity.

Manipulation of these costimulatory pathways to promote antiviral or antitumoural immunity as well as for the control of autoimmune diseases or transplant rejection has been proposed as therapeutic strategy and is addressed by either activation or blockade of the corresponding signalling elements. Particularly agonists of OX40 and CD27, mainly monoclonal antibodies, have been developed and are currently being tested in preclinical models and clinical trials to enhance the efficacy of antitumour immunotherapies [14,15]. Due to their critical role for the development of primary as well as memory responses against viral infections [16,17], their use as adjuvants in conventional vaccination has also been proposed [18] and both secreted OX40L- and CD70-based constructs have been shown to enhance immune responses to an HIV-1 DNA vaccine in a murine model [19].

In ruminants, vaccination can be used to prevent infections by a set of parasitic, viral, and bacterial pathogens [20,21] that collectively produce severe economic and social burden worldwide [22]. These include important animal diseases such as bluetongue, peste des petits ruminants or foot and mouth disease, as well as zoonotic diseases like rift valley fever [23,24,25,26]. Improved vaccine designs for these diseases are being developed and these include the development of specific immunomodulators as coadjuvants. As costimulatory signalling pathways in the TNF/TNFR superfamilies in sheep are still uncharacterised, its understanding is of critical relevance to develop their use as immunity modulators.

In this report, we studied the potential orthologues of OX40L-OX40 and CD70-CD27 molecules in sheep, providing evidence for the first time of their interaction and activity using cloned and expressed recombinant proteins. Moreover, we generated recombinant non-replicative human adenoviruses expressing immunostimulatory ligands for both receptors. Non-replicative human adenoviral vectors have been successfully used as efficient in vivo protein delivery systems [27,28,29] and their use is particularly suited for veterinary medicine, as the host lacks pre-existing immunity to this human vector. Additionally, these vectors are safe, present low toxicity, transduce a broad spectrum of host species, and are relatively easy to manipulate and distribute [30]. This work identifies the capacity of soluble ovine OX40L and CD70 forms to activate cells expressing their cognate receptor and stimulate ovine T cell responses, which points to their potential use as adjuvant for vaccination strategies in ruminants

## 2. Materials and Methods

### 2.1. Cells and Viruses

HEK293 cells (ATCC CRL-1573) and its derivative as well as Vero cells (ATCC CCL-81) were grown in Dulbecco’ minimal essential medium (DMEM, Gibco, Dublin, Ireland), supplemented with 10% Foetal Bovine Serum (FBS) (Sigma-Aldrich, Saint Louis, MO, USA), 2 mM L-glutamine, 1% 100× non-essential amino-acids (AANE), 1 mM sodium pyruvate and 100 U/mL Penicillin/100 μg/mL Streptomycin (all from Thermofisher Scientific, Waltham, MA, USA). The HEK293 derived reporter cell line HEK293/pr(IFNβ)-GFP that contains the enhanced Green Fluorescent Protein (eGFP) coding gene under the control of the human IFNβ promoter were kindly provided by Dr. R.E. Randall (St. Andrews University, Scotland, UK). The *Trichoplusia ni*-derived insect cell line Hi5 (Thermofisher Scientific, Waltham, MA, USA) was grown in TC-100 medium (Sigma-Aldrich, Saint Louis, MO, USA) supplemented with 10% FCS for adherent cell culture and in ExpressFive serum free medium (Gibco, Dublin, Ireland) for suspension culture and protein expression experiments.

Stocks of recombinant baculoviruses vBAC-*Oa*OX40L and vBAC-*Oa*CD70 were amplified twice on Hi5 cells to generate working stocks for protein expression procedures and stored at 4 °C until used. The recombinant adenoviruses Ad5-*Oa*OX40L and Ad5-*Oa*CD70 were amplified by sequential rounds of growth on HEK293 cells and final stocks were purified and titrated using standard protocols as described before [31] and stored at −70 °C until used.

### 2.2. Cloning and Generation of Recombinant Viruses

The putative *Ovis aries* (*Oa*) *OX40* (*TNFRSF4*), *OX40L (TNFSF4), CD27* (*TNFRSF7*) and *CD70 (TNFSF7)* were identified using blastp and/or tblastn searches using previously annotated orthologues from other mammalian species as baits and retrieved from different databases. The accession numbers for these molecules are OX40 (Uniprot W5P810), OX40L (Uniprot W5PZ67), CD27 isoform X1 (Genbank XP_004006990), and CD70 (Uniprot W5P639). For sequence alignments shown in Supplementary Figure S1, orthologues from additional mammalian species were retrieved (accession numbers on figure legend) and aligned online using Clustal omega software (https://www.ebi.ac.uk/Tools/msa/clustalo/).

The DNA sequences encoding full length *Oa*OX40 and *Oa*CD27 were optimised for expression in mammalian cells and synthesised in vitro (GenScript, Piscataway, NJ, USA). For transient expression assays, these were subcloned into plasmid pcDNA3.1V5HisA (Invitrogen, Waltham, MA, USA). Similarly, all DNA sequences for the recombinant *Oa*OX40L and *Oa*CD70 constructs were assembled in silico and optimised coding sequences designed and synthesised in vitro as above. The details of the relevant elements found in these constructs are shown in Supplementary Figure S1 and correspond to an N- terminal signal peptide derived from insulin, followed by the region encoding Fc (residues P245-K473) form the immunoglobulin gamma-1 chain from *Ovis aries* (Accession number CAA49451), an isoleucine trimerization domain [32] and the predicted extracellular domain of the corresponding TNFSF protein. These constructs were then subcloned into either pSIREN_EF1 plasmid for the generation of recombinant adenoviruses or pOET3 plasmid (Oxford Expression Technologies, Oxford, UK) for the generation of recombinant baculoviruses. The cloning strategies and coding sequences of all plasmids employed is available upon request.

Recombinant baculoviruses were obtained using the flashBAC GOLD system (Oxford Expression Technologies, Oxford UK) following the manufacturer’s instructions. Briefly, the plasmids pOET3-*Oa*OX40L or pOET3-*Oa*CD70 were cotransfected with a linearised DNA corresponding to a modified baculovirus genome optimised for expression of secreted proteins in Hi5 insect cells and recombinant baculoviruses recovered in a single step from the supernatants and stored at 4 °C.

The procedure to obtain recombinant adenoviruses has been described in detail before [31]. Briefly, the donor plasmids pSIREN-*Oa*OX40L or pSIREN-*Oa*CD70 were used to transfer the gene of interest to a commercial Acceptor Vector pLP-Adeno-X-PRLS (Clontech, Mountain View, CA, USA) by Cre-loxP mediated recombination. The resulting adenoviral vector DNAs were linearised and transfected into HEK293 cells that provide in *trans* the adenoviral replicative capacity and allow the recovery of the replication defective adenoviruses in the supernatants of the transfected cells.

### 2.3. Protein Detection by Western Blot

Vero cells were seeded in M-24 well plates and infected with Ad5-*Oa*OX40L, Ad5-*Oa*CD70, and Ad5-DsRed (empty recombinant adenovirus used as negative control) at a multiplicity of infection (moi) of 1. Twenty-four hours post-infection the supernatants and cells were harvested separately. They were resuspended in Laemmli buffer and analysed by Western blot with a polyclonal serum. Briefly, equivalent amounts of whole cell extracts (or supernatants) were electrophoresed on SDS-10% polyacrylamide gels and transferred to nitrocellulose membranes. The membranes were incubated with the anti-sheep-Fc (Bethyl, Montgomery, TX, USA) or anti-tubulin mouse IgG1 monoclonal antibody (Sigma-Aldrich, Saint Louis, MO, USA) and then with a peroxidase-labelled anti-mouse serum (GE Life Sciences, Marlborough, MA, USA). Protein bands were detected with the ECL system (GE Life Sciences, Marlborough, MA, USA) according to the manufacturer’s recommendations.

### 2.4. Purification of Recombinant Ovine CD70 (r*Oa*CD70) and OX40L (r*Oa*OX40L) Proteins

For the purification of recombinant protein constructs, Hi5 cells were grown to high densities in 200 mL batches of suspension cultures in serum free medium. The cells were infected at a multiplicity of infection of approximately 10 pfu/cell. At 72 h post infection, supernatants containing recombinant proteins were harvested by sequential centrifugation at 500 and 3000 g for 15 min to remove cells and cellular debris. The clarified media were concentrated using a Minimate Tangential Flow Filtration System (PALL) and diafiltered into 20 mM phosphate buffer pH 7.0. The recombinant proteins were then subjected to immunoaffinty chromatography using prepacked GE healthcare HiTrap Protein G-sepharose columns (Thermofisher Scientific, Waltham, MA, USA) following the manufacturer’s instructions and dialysed into 0.2 M Hepes, 1.5 M NaCl, pH 7.4 buffer containing 0.01% sodium azide. The purified proteins were analysed by Coomassie blue-stained SDS-PAGE and quantified using a standard BCA assay and stored at −70 °C for use.

### 2.5. Immunofluorescence Microscopy

Semiconfluent HEK293 or HEK293/pr(IFNβ)-GFP cell monolayers seeded in coverslips were transfected using Mirus *Trans*iT-LT1 transfection reagent (Mirus Bio LLC, Madison, WI, USA) following the manufacturer’s instructions. At the corresponding time, 10 coverslips per sample were fixed using a 4% paraformaldehyde solution, washed three times with PBS, and blocked with Dako antibody diluent (Dako, #S3022) for 1 h at room temperature (RT). Proteins were detected by indirect immunofluorescence using primary antibody [mouse anti-V5 (Sigma-Aldrich, Saint Louis, MO, USA) diluted in Dako antibody diluent and incubated overnight at 4 °C, and anti-mouse Alexa-647 or anti-sheep-Fc-Alexa 488 (Thermofisher Scientific, Waltham, MA, USA) secondary antibodies for 1 h at RT. Nuclei were counterstained using 4′,6-diamidino-2-phenylindole DAPI (Sigma-Aldrich, Saint Louis, MO, USA). Cells were washed and coverslips mounted using Prolong Gold antifade reagent (Invitrogen, Waltham, MA, USA). Images were captured using an Olympus CKX41 fluorescence microscope for GFP induction in HEK293/pr(IFNβ)-GFP or a LSM 880 confocal microscope for colocalisation analysis. Image analysis was performed with the ImageJ software (http://rsbweb.nih.gov/ij/ US National Institutes of Health). For r*Oa*OX40L or r*Oa*CD70 pixel colocalisation analysis with *Oa*OX40 or *Oa*CD27 signal, respectively, the percentage of overlapping positive signal per cell was determined as follows: confocal z-stack images (step 0.3–0.5 μm) of transfected cells were captured for each channel, cell mask obtained [33], positive signal for each channel was determined with the ImageJ threshold tool, and percentage of overlapping pixels in cell masks for each z-plane determined with the ImageJ image calculator tool (“AND” operation).

### 2.6. PBMC Isolation and T Cell Enrichment

Sheep PBMC were obtained from healthy donor ewes housed at the *“Departamento de Reproducción Animal at the INIA”* (Madrid) by standard density gradient centrifugation methods [29]. For proliferation assays, T cell enrichment was performed with nylon-wool columns [34,35]. Briefly, PBMC were incubated for 45 min on nylon wool column, and the initial column effluent, enriched in T cells, was used in the subsequent experiments. T cell enrichment was verified with anti-CD3 staining (clone CD3-12) and flow cytometry. Enriched T cell fractions typically contained >80% CD3^+^ cells. Enriched T cell fractions and PBMC were cultured in RPMI (Lonza, Basel, Switzerland) supplemented with 10% FBS (Sigma-Aldrich, Saint Louis, MO, USA), 2 mM L-glutamine, 10 mM HEPES, 1% 100× non-essential amino-acids, 1 mM sodium pyruvate, 100 U/mL penicillin/100 μg/mL streptomycin, and 50 nM 2-mercaptoethanol (all from Thermofisher Scientific, Waltham, MA, USA) [36].

### 2.7. CFSE Proliferation Assays

To assess the functionality of the soluble forms produced after Ad5-*Oa*OX40L or Ad5-*Oa*CD70 cell infection, enriched sheep T cells derived from *n* = 5 animals were independently labelled with CellTrace CFSE (Thermofisher Scientific, Waltham, MA, USA) as described by the manufacturer’s instruction. Enriched T cells (2 × 10^6^ per well) were then cultured in 24-well plates for 4 days in presence of supernatants from Ad5-DsRed-, Ad5-*Oa*OX40L-, or Ad5-*Oa*CD70-infected Vero cells (20% (v/v) supernatant). Cells were then acquired on a FACSCalibur flow cytometer (Becton Dickinson) and data analysed with FlowJo software (Tree Star Inc.).

### 2.8. Intracellular Cytokine Staining and Flow Cytometry

To induce IFN-γ production, PBMC were stimulated overnight with 1.25 μg/mL concanavalin-A (ConA) (Sigma-Aldrich, Saint Louis, MO, USA) and supernatant obtained from Ad5-DsRed-, Ad5-*Oa*OX40L-, or Ad5-*Oa*CD70-infected Vero cells. Brefeldin-A (5 μg/mL) (Biolegend, San Diego, CA, USA) was added in the last 4 h of incubation. Cells were then labelled as described in [29,35] with anti-sheep-CD4-FITC (clone 44.38), anti-sheep-CD8-PE (clone 38.65), and anti-bovine-IFN-γ-Alexa 647 (clone CC302) antibodies (all from Bio-Rad, Madrid, Spain). Samples were acquired on a FACSCalibur flow cytometer (Becton Dickinson) and data analysed with FlowJo software (Tree Star Inc.). Percentage of IFN-γ^+^ cells within the CD4^+^ or CD8^+^ T cell compartment were measured. Isotype and fluorescence minus one-channel controls were used for gating strategy [37].

To assess CD27 expression in sheep PBMC subpopulations, PBMC obtained from healthy donor ewes (*n* = 15) were co-stained with cross-reactive anti-human/mouse/rat CD27 antibody (clone LG-3A10 from Biolegend) and either anti-ovine CD4, -ovine CD8, -ovine CD335 (all three from Bio-Rad), or -bovine B cell marker (Kingfisher Biotech) as detailed elsewhere [35]. Gating for CD27^+^ events was set using antibody isotype and fluorescence minus-one channel controls. Data were acquired on a FACSCalibur flow cytometer (Becton Dickinson) and analysed with FlowJo software (Tree Star Inc.).

### 2.9. Statistical Analyses

Data handling and statistical analyses was performed using Prism 6.0 software (GraphPad Software Inc. San Diego, CA, USA). Statistical tests used to compare data are indicated in the figure legend.

### 2.10. Ethics Statement

This study was carried out in strict accordance with the recommendations in the guidelines of the Code for Methods and Welfare Considerations in Behavioural Research with Animals (Directive 86/609EC; RD1201/2005) and all efforts were made to minimise suffering. Experiments were approved by the Committee on the Ethics of Animal Experiments (CEEA) (Permit number: 10/142792.9/12) of the Spanish Instituto Nacional de Investigación y Tecnología Agraría y Alimentaria (INIA) and the Comisión de ética estatal de bienestar animal (Permit numbers: CBS2012/06 and PROEX 228/14).

## 3. Results

### 3.1. Purification of Soluble Secreted Recombinant Ovine Proteins, r*Oa*OX40L and r*Oa*CD70

**Figure 1:**
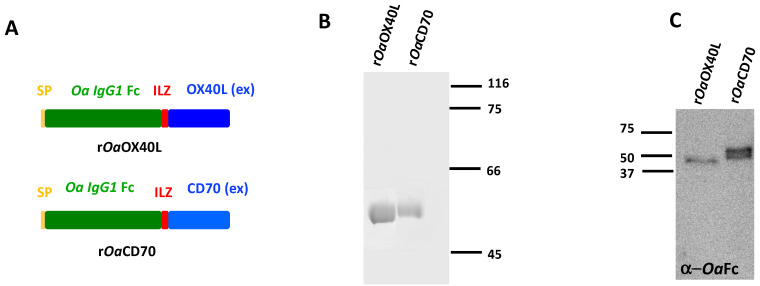
Purification of recombinant proteins r*Oa*OX40L and r*Oa*CD70. (**A**) Schematic representation of the protein constructs expressed by the recombinant baculoviruses and adenoviruses. They include an insulin derived signal peptide (SP) the ovine (Oa) *IgG1* Fc domain, the short isoleucine trimerization domain (ILZ) followed by either the predicted extracellular domain from ovine OX40L or ovine CD70. (**B**) Coomassie-blue stained SDS-PAGE showing immunoaffinity purified recombinant proteins. Molecular Weigth Marker in kilodaltons (kDa) are shown on the right. (**C**) Western blot analysis of the purified proteins detected by an anti-ovine Fc antibody. MWM (kDa) are shown on the left.

The potential ovine orthologues of OX40L-OX40 and CD70-CD27 were identified using blastp and tblastn searches and found to contain all the characteristic domains of TNF and TNFR superfamilies, respectively (Supplementary Figure S1). In order to characterise the ovine OX40L and CD70 proteins, we first purified recombinant versions of both proteins. To this end, we constructed recombinant baculoviruses expressing the predicted extracellular domain of each ligand fused to an isoleucine zipper (ILZ) trimerization motif [32] and an ovine IgG Fc domain (Ov Fc). A secretion signal (SP) was incorporated at the N-terminus of each construct to allow secretion into the extracellular medium (Figure 1A). Recombinant proteins of the expected molecular weights (48 kDa) were purified by immunoaffinity chromatography from the supernatants of the corresponding baculovirus-infected insect cells (Figure 1B). Western blot analysis using anti-ovine Fc antibodies confirmed the identity of the recombinant proteins (Figure 1C).

### 3.2. The r*Oa*OX40L and r*Oa*CD70 Proteins Bind to Cells Expressing Their Predicted Cognate Receptors

**Figure 2:**
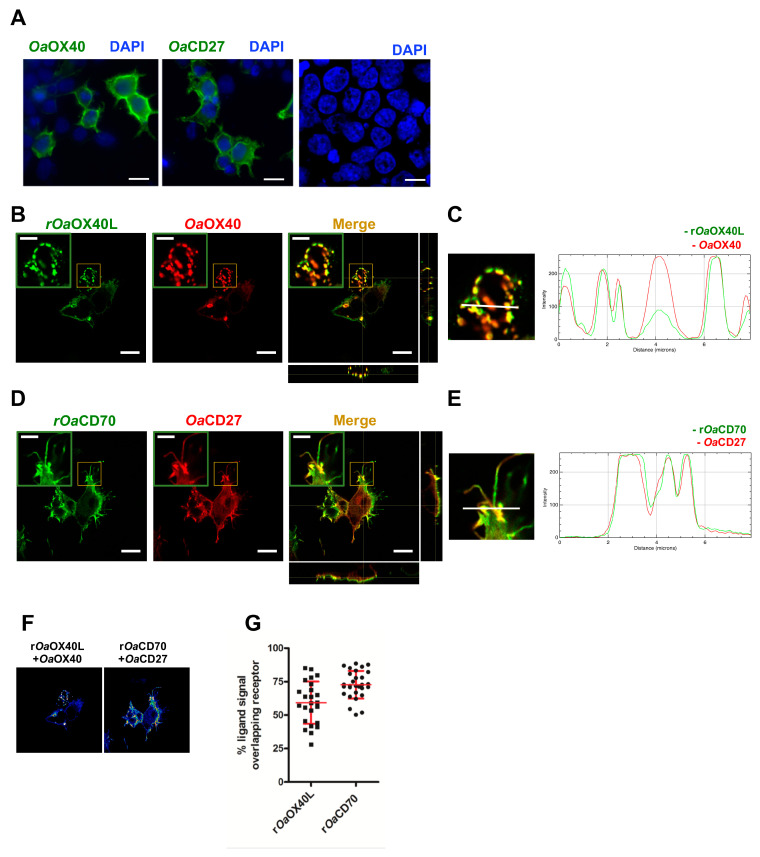
Colocalisation of the ovine OX40L and CD70 ligands with their cognate receptors. (**A**) Immunolocalisation of *Oa*OX40 and *Oa*CD27 in transfected HEK293 cells detected by anti-V5 tag antibody and counterstained with DAPI. Cells expressing (**B**) *Oa*OX40 or (**D**) *Oa*CD27, were incubated with 10 μg of (**B**) purified r*Oa*OX40L or (**D**) purified r*Oa*CD70 for 15 min before fixation and immunofluorescence. *Oa*OX40 and *Oa*CD27 expression was detected using anti-V5 tag antibodies (red); r*Oa*OX40L and r*Oa*CD70 presence were detected with an anti-ovine Fc antibody (green). (**B**,**D**) Insets show detail of colocalisation in each case. Merge and orthogonal projection were obtained with ImageJ software. Scale bar = 10 μm, indent scale bar = 4 μm. In panel (**C**) and (**E**), an enlarged image of corresponding insets in Merge and fluorescence intensity profiles for the indicated white lines on the images are shown. (**F**) ImageJ Image calculator function was used to evaluate colocalisation of ligand signal with its receptor. Representative images of colocalisation analysis in a Z-plane (presented as 16LUT signal) are shown. The ImageJ calculator tool was used for pixel colocalisation for the fluorescence channels of ligands (r*Oa*OX40L or r*Oa*CD70) and their respective receptors (r*Oa*OX40 or r*Oa*CD27). (**G**) The percentage of recombinant ligand signals colocalising with their receptors in (**F**) was analysed in 25–40 cells for each condition. Mean ± SD are indicated for each condition.

We next transfected plasmids bearing C-terminally V5 tagged versions of the predicted full-length ovine receptors for *Oa*OX40L (*Oa*OX40) and *Oa*CD70 (*Oa*CD27) in HEK293 cells. Both receptors were expressed and showed a pattern consistent with their expected location at the plasma membrane upon immunofluorescence analyses (Figure 2A). To detect binding of the recombinant r*Oa*OX40L and r*Oa*CD70 proteins, these were incubated with the cells at 4 °C for a short period and immunofluorescence detection using an anti-ovine Fc tag antibody after fixation was performed. While recombinant proteins were not detected on non-transfected cells, r*Oa*OX40L was found to bind specifically to *Oa*OX40-expressing cells (Figure 2B,C), and r*Oa*CD70 to *Oa*CD27-expressing cells (Figure 2D,E). In both cases, a punctate pattern on the cell surface corresponding to the ligands was detected which overlapped with that detected for the receptors. Fluorescence intensity profiles in sections indicated by white lines on the images (Figure 2C,E) show an almost identical profile in both fluorophores. Pixel colocalisation studies (Figure 2F) showed an average percentage of ligand sharing pixel localisation with its putative receptor of 59.3 ± 15.9% for the r*Oa*OX40L/*Oa*OX40 and 72.8 ± 10.3% for the r*Oa*CD70/*Oa*CD27 pair, respectively (Figure 2F,G).

### 3.3. Recombinant Adenoviruses Express and Secrete r*Oa*OX40L and r*Oa*CD70 Proteins

**Figure 3:**
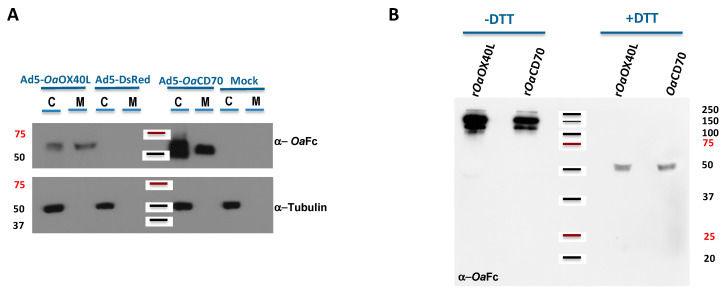
Expression and secretion of r*Oa*OX40L and r*Oa*CD70 using recombinant adenoviruses. (**A**) Vero cell monolayers were not infected (mock) or infected with the recombinant Ad5-*Oa*OX40L, Ad5-*Oa*CD70, or the control Ad5-DsRed viruses as indicated. At 48 hpi, the cells (C) and media (M) were harvested separately and equivalent amounts analysed by Western blot for the presence of ovine Fc-bearing proteins (upper panel) or tubulin (lower panel). The r*Oa*OX40L and r*Oa*CD70 molecules are readily detected both in the cell extracts and in the media, showing that the proteins are secreted from the infected cells. Absence of cellular tubulin in these media was used as a control of the fractionation procedure. (**B**) The media containing r*Oa*OX40L and r*Oa*CD70 from panel A were analysed in the presence or absence of DTT as indicated by Western blot to detect the formation of disulfide linked oligomers in the recombinant proteins. The position of MWM (kDa) is indicated in both panels.

**Figure 4:**
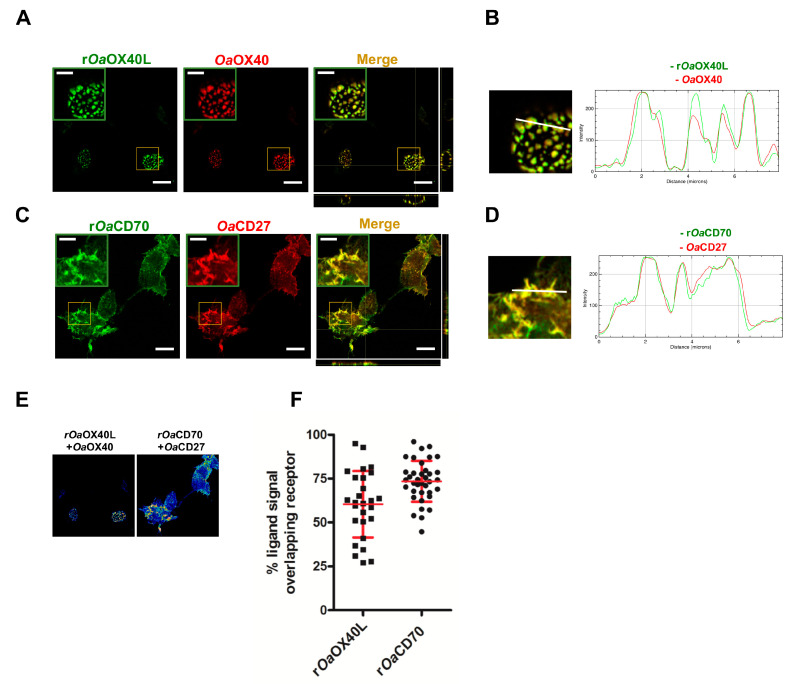
The secreted r*Oa*OX40L and r*Oa*CD70 expressed from recombinant adenovirus-infected cells colocalise with their cognate ovine receptors. Cells expressing (**A**) *Oa*OX40 or (C) *Oa*CD27, were incubated with conditioned media from cells infected with (**A**,**B**) Ad5-*Oa*OX40L (r*Oa*OX40L) or (**C**,**D**) Ad5-*Oa*CD70 (r*Oa*CD70) for 15 min before fixation and immunofluorescence. r*Oa*OX40L and r*Oa*CD70 were detected with an anti-Fc antibody (green); *Oa*OX40 and *Oa*CD27 expression was detected using anti-V5 tag antibodies (red). (**B**,**D**) Insets show detail of colocalisation for each case. Merge and orthogonal projections were obtained with ImageJ software. Scale bar = 10 μm, indent scale bar = 4 μm. In panel (**B**) and (**D**), an enlarged image of corresponding insets in Merge and fluorescence intensity profiles for the indicated white lines on the images are shown. (**E**) Representative images of colocalisation analysis in a Z-plane (presented as 16LUT signal) are shown. The ImageJ calculator tool was used for pixel colocalisation for the fluorescence channels of ligands (r*Oa*OX40L or r*Oa*CD70) and their respective receptors (r*Oa*OX40 or r*Oa*CD27). (**F**) The percentage of (**E**) adenovirus-produced ligands signal colocalising with their receptors was analysed in 25–40 cells for each condition. Mean ± SD are indicated for each condition.

To allow sustained expression of the secreted ligands as potential immunomodulators in vaccinated hosts, we generated recombinant human adenoviruses Ad5-*Oa*OX40L and Ad5-*Oa*CD70 bearing the same constructs as above. Thus, extracts from Vero cells infected with the recombinant viruses were analysed by Western blot. Specific bands corresponding to the predicted weights for the r*Oa*OX40L and r*Oa*CD70 protein constructs (48 kDa) were detected both in the cell extracts as well as in the culture media fractions of the corresponding samples, but not in those of mock-infected cells or cells infected with a control adenovirus (Figure 3A). The intracellular protein tubulin was only detected in the cell extract samples (C) and not in the supernatants (M), showing that cells were not lysed during the harvest procedure. Therefore, the generated adenoviruses express and secrete the recombinant *Oa*OX40L and *Oa*CD70 proteins. Analysis of the samples from the culture media in the presence or absence of the reducing agent DTT showed that both proteins formed disulphide-bridge mediated oligomers, possibly trimers, under native conditions (Figure 3B). Conditioned media containing r*Oa*OX40L and r*Oa*CD70 proteins were incubated with HEK293 cells transfected with the *Oa*OX40 and *Oa*CD27 receptors as above and binding of the ligands was detected only in those cells expressing their respective cognate receptors (Figure 4A,C). Fluorescence intensity profiles in sections indicated by white lines on the images showed an almost identical profile in both fluorophores (Figure 4B,D) and quantitative analysis in cells of ligand/receptor pair colocalisation showed 60.4 ± 18.9% for the r*Oa*OX40L/*Oa*OX40 and 73.5 ± 11.7% for the r*Oa*CD70/*Oa*CD27 pair, respectively (Figure 4E,F).

### 3.4. Ovine OaOX40 and OaCD27 Act as Cognate Signalling Receptors for rOaOX40L and rOaCD70

**Figure 5:**
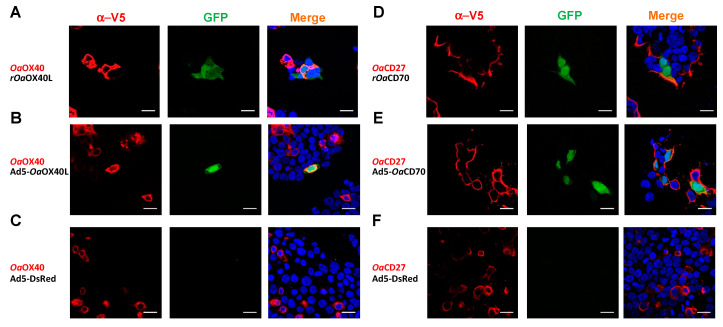
The r*Oa*OX40L and r*Oa*CD70 specifically elicit signalling through their cognate receptors *Oa*OX40 and *Oa*CD27. HEK293-pr(IFNB)-GFP cells were transfected with, (**A**–**C**) *Oa*OX40-V5 and (**D**–**F**) *Oa*CD27-V5. At 24 h post transfection, the cells were stimulated with 10 μg of purified (**A**) *rOa*OX40L or (**D**) *rOa*CD70 proteins, or equivalent amounts of conditioned media from Ad5-*Oa*OX40L (**B**) *rOa*OX40L, (**E**) Ad5-*Oa*CD70, or (**C**,**F**) Ad5-DsRed infected Vero cells proteins in conditioned media from Ad5-*Oa*OX40L, Ad5-*Oa*CD70, or Ad5-DsRed infections. At 16 h post stimulation, the cells were fixed and an immunofluorescence using anti-V5 (red) to detect the transfected receptors was performed. GFP (green) activity was detected by fluorescence microscopy and DAPI was used to counterstain cells to nuclei (blue). Bars = 20 µm.

In order to determine whether the secreted *Oa-*OX40L and *Oa-*CD70 can induce signalling through their cognate ovine receptors, we took advantage of the HEK293/pr(IFN)-GFP reporter cell line, which expresses GFP under the control of the human IFN promoter [pr(IFN)], which contains several NF-κB sites which are inducible through the NF-κB signalling pathway employed by non-death domain containing TNFRs. In the first place, we transfected the reporter cells to express the *Oa*OX40 (Figure 5A–C) or *Oa*CD27 (Figure 5D–F) proteins. At 24 h post transfection, the cells were stimulated with 10 μg of purified r*Oa*OX40L (Figure 5A) or r*Oa*CD70 (Figure 5D) proteins, respectively, and GFP expression was assessed at 16 h poststimulation by fluorescence microscopy. In the absence of ligand proteins from the cognate pair, no GFP expression was detected (Figure 5C,F). In contrast, both recombinant ligands were able to elicit GFP expression on cells expressing their cognate receptor, showing that the ovine ligands can induce specific signalling through their corresponding receptors (Figure 5A,D). Likewise, media from Vero cells infected with Ad5-*Oa*OX40L (Figure 5B) or Ad5-*Oa*CD70 (Figure 5E) but not those media from cells infected with a control adenovirus (Ad5-DsRed) (Figure 5C,F) were able to induce GFP expression specifically in the reporter cells transfected with the corresponding receptors.

**Figure 6:**
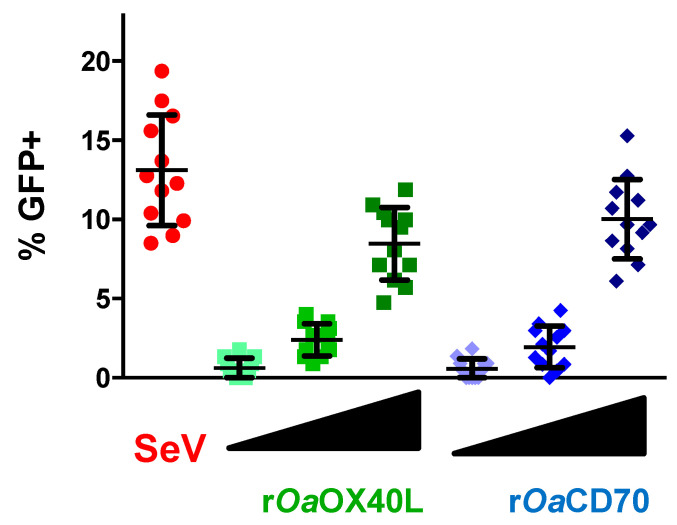
Dose dependent induction of signalling by r*Oa*OX40L and r*Oa*CD70 ligands. HEK-293/prIFNB-GFP cells were transfected with plasmids to express the receptors *Oa*OX40 or *Oa*CD27 and stimulated at 24 h post transfection with three equivalent and increasing doses (1×, 2×, and 4×) of conditioned media from Ad5-*Oa*OX40L (r*Oa*OX40L) or Ad5-*Oa*CD70 (r*Oa*CD70) infected Vero cells. As a positive control of signalling induction, untransfected cells were infected with Sendai virus (SeV) at a multiplicity of infection (moi) of 1 pfu/cell for 16 h. GFP expressing cells were counted on an immunofluorescence microscope on 12 randomly chosen fields and % of GFP expressing cells for each field are plotted. Mean ± SD for each condition are indicated with bars.

We next assessed GFP expression in reporter cells that were transfected with the receptors and incubated with increasing amounts of the r*Oa*OX40L or r*Oa*CD70 containing conditioned media. GFP expression was evaluated in 12 different random fields per condition and represented as percentage of cells expressing GFP per field (Figure 6). As shown, a clear dose-dependent increase in GFP^+^ cells was observed in the case of each ligand–receptor pair, supporting the specificity of the observed interaction and signalling activation.

### 3.5. Detection of Endogenous CD27 Protein in Ovine Peripheral Blood Mononuclear Cells

**Figure 7:**
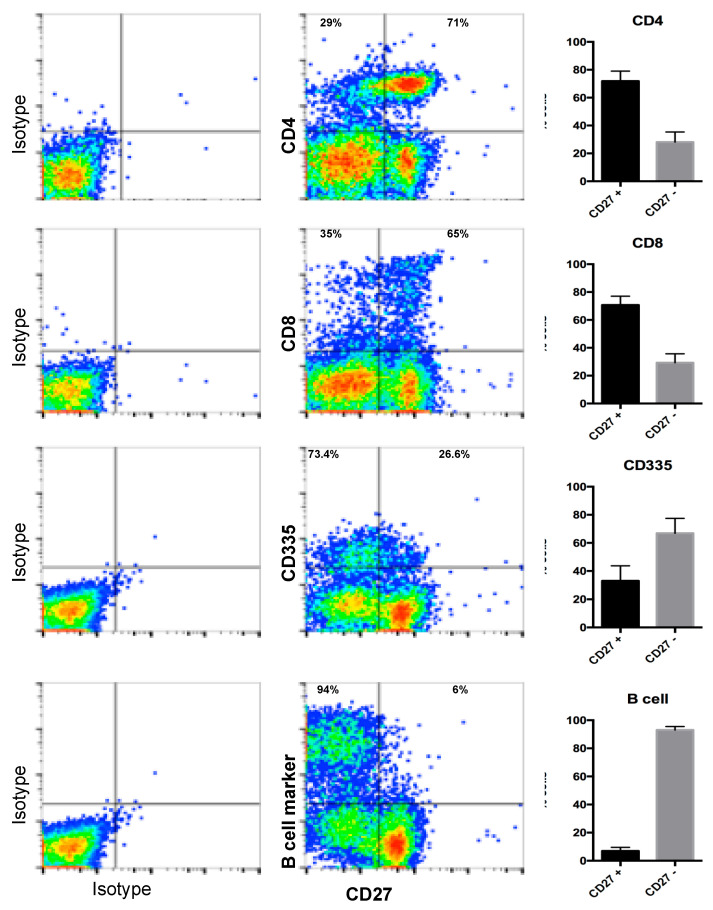
CD27 expression on ovine PBMC populations. Ovine PBMC, derived from *n* = 15 animals were costained for CD27 and CD4, CD8, CD335, or B cell markers. Gates were set using the corresponding isotype controls. Isotype control staining and representative dot plots for CD27 staining and CD4; CD8; CD335 or B cell marker are shown. Bar charts show the mean (±SD) percentage of CD27^+^ and CD27^-^ cells in the CD4/CD8/CD335/B cell marker gates (upper quadrants) in donor sheep PBMC.

We next addressed the expression of the OX40 and CD27 receptors in ovine immune cells. To the best of our knowledge, no commercial antibodies have been reported to detect OX40 or CD27 in sheep. We used HEK293 cells transfected with the receptor expressing constructs previously described in flow cytometry assays with commercial antibodies generated against the murine OX40 or the murine CD27. Of these, the anti OX40 antibody is known to only react with the murine protein and in our experiment, it did not cross-react with the ovine molecule in spite of high expression of the receptor on the transfected cells as assessed by anti V5 tag flow cytometry staining (not shown). By contrast, the anti-mouse CD27 antibody which is known to react with mouse, rat, and human orthologues, specifically recognised the ovine CD27 on transfected cells. We then used this antibody to detect endogenous CD27 expression on ovine PBMCs obtained from donor sheep. As shown on Figure 7, ovine CD27 is expressed on the majority of CD4^+^ (71.8 ± 7.2%) and CD8^+^ (70.7 ± 6.4%) T cells and in a subset of NK cells (33.1 ± 10.4%). CD27 was only present at low levels on circulating B cells (7 ± 2.5%).

### 3.6. OaOX40L and OaCD70 Stimulate Ovine T Cell Proliferation and IFN-γ Production

**Figure 8:**
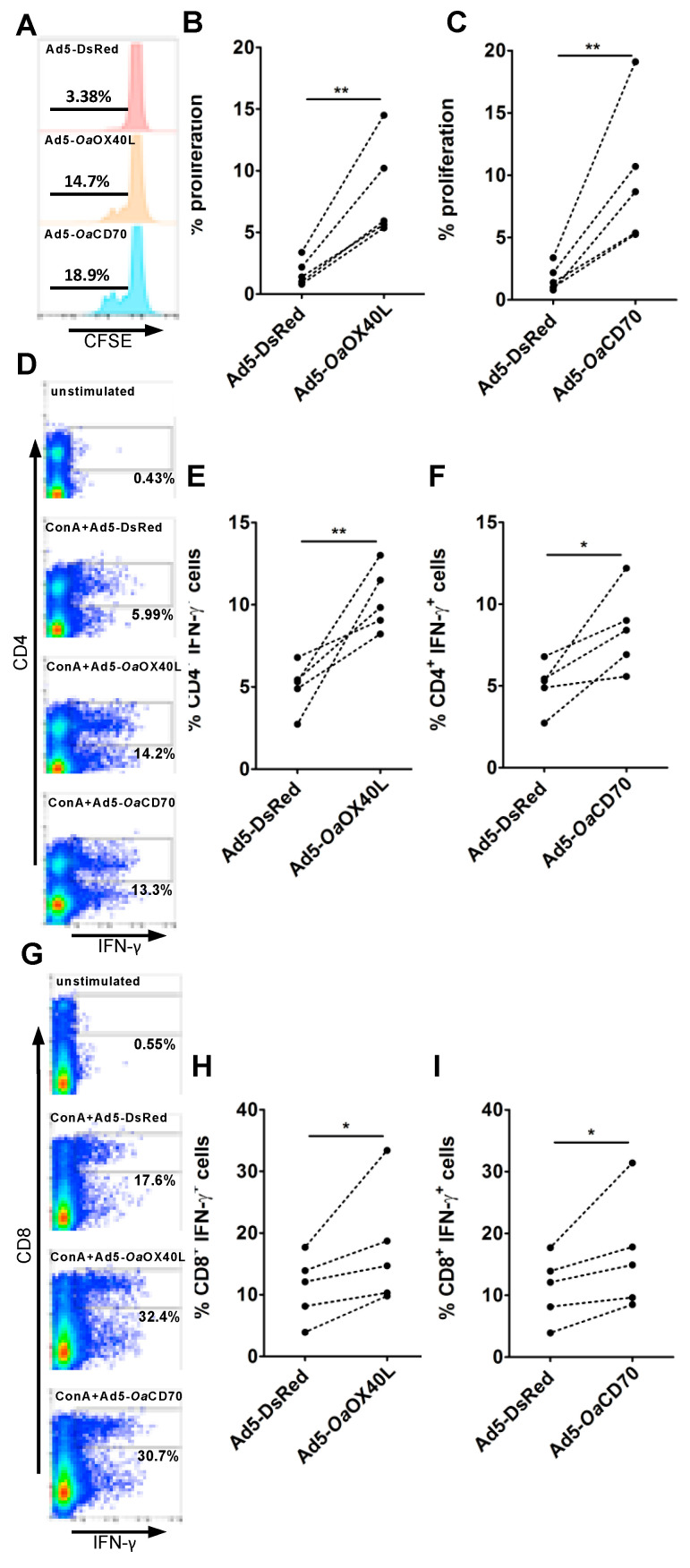
The r*Oa*OX40L and r*Oa*CD70 ligands promote sheep T cell proliferation and IFN-γ production. (**A**–**C**) An enriched T cell fraction was obtained from sheep (*n* = 5) PBMCs, labelled with CFSE and cultured for 4 days with equivalent amounts of conditioned media from Ad5-*Oa*OX40L-, Ad5-*Oa*CD70-, or Ad5-DsRed-infected Vero cells. Cell fluorescence was then acquired by flow cytometry. (**A**) Representative flow cytometry histograms show the reduction in CFSE fluorescence proportional to cell proliferation. (**B**,**C**) Proliferation induced by (**B**) Ad5-*Oa*OX40L or (**C**) Ad5-*Oa*CD70 conditioned media in the T cell fractions obtained from five individual donor sheep. ** *p* < 0.01 Paired Student’s *t*-test (Ad5-DsRed vs. Ad5-*Oa*OX40L or Ad5-*Oa*CD70). (**D–I**) Sheep PBMC were stimulated with ConA and cultured with media as indicated for 18 h. IFN-γ production was then evaluated by intracellular cytokine staining and flow cytometry in (**D–F**) CD4^+^ and (**G–I**) CD8^+^ T cells. (**D**,**G**) Representative flow cytometry dot-plots showing IFN-γ production in (**D**) CD4^+^ and (**G**) CD8^+^ T cells for unstimulated PBMC and ConA-stimulated PBMC in the presence of Ad-DsRed, Ad-*Oa*OX40L, or Ad-*Oa*CD70 conditioned media. Indicated percentages represent the number of positive cells in the CD4^+^ or CD8^+^ T cell compartment. (**E**,**F**,**H**,**I**) IFN-γ production in (**E**,**F**) CD4^+^ and (**H**,**I**) CD8^+^ T cells induced by (**E**,**H**) Ad5-*Oa*OX40L- or (**F**,**I**) Ad-*Oa*CD70-conditioned media in PBMC obtained from five individual donor sheep. * *p* < 0.05; ** *p* < 0.01 Paired Student’s *t*-test (Ad-DsRed vs. Ad5-*Oa*OX40L or Ad5-*Oa*CD70).

Engagement of *Oa*OX40 or *Oa*CD27 by their ligand (*Oa*OX40L or *Oa*CD70, respectively) can stimulate effector T cell activity in human and mice [38,39,40]. To evaluate whether this is the case in sheep, the enriched T cell fraction isolated from sheep PBMC was cultured in presence of conditioned media containing r*Oa*OX40L or r*Oa*CD70 recombinant proteins expressed by the respective adenovirus and cellular proliferation tested using CFSE labelling (Figure 8A–C). Control conditioned media did not have any stimulatory effect on proliferation while supernatants from either Ad5-*Oa*OX40L- or Ad5-*Oa*CD70-infected Vero cells stimulated T cell proliferation in all tested donor sheep. To further confirm the co-stimulatory capacity of the r*Oa*-OX40L and r*Oa*-CD70 soluble proteins, PBMC were stimulated with ConA and IFN-γ production measured in CD4^+^ and CD8^+^ T cells (Figure 8D–I). The presence of r*Oa*-OX40L or r*Oa*-CD70 in the culture medium increased the number of CD4^+^ (Figure 8D–F) and CD8^+^ (Figure 8G–I) T cells producing IFN-γ in PBMC from all tested sheep. These data show that both r*Oa-*OX40L and r*Oa-*CD70 can stimulate T cell activity in sheep, an effect that is probably mediated by activation through their cognate ovine receptors described above.

## 4. Discussion

In this report, we identified and characterised for the first time the ovine costimulatory molecules of the TNFR superfamily OX40 and CD27 as well as their cognate ligands, OX40L and CD70. These receptors were found to contain the characteristic elements of TNFRs including the extracellular cysteine rich ligand binding domains followed by a predicted transmembrane region and a cytoplasmic tail which, like their human or murine counterparts lack death domains and therefore signal through direct binding to TNF receptor associated factor (TRAF) binding proteins [12,41]. Transient expression assays allowed us to determine that the ovine *Oa*OX40 or *Oa*CD27 proteins could be found at the plasma membrane on transfected cells and colocalised with exogenously added purified *rOa*OX40L or *rOa*CD70 molecules, respectively. Moreover, addition of their predicted ligands induced NF-κB mediated signalling in a specific and dose dependent manner. Altogether, these results suggest that the ovine ligands bind specifically to their ovine receptors on the cell surface, inducing NF-κB signalling. This shows that the costimulatory signalling axes represented by OX40L-OX40 and CD70-CD27 are conserved and functional in the ovine species and that the recombinant ligands obtained here can effectively elicit biological activity.

With the aim of developing tools for the immunomodulation of these costimulatory pathways, we generated biologically active agonist recombinant forms of the ovine ligands. The oligomerisation of TNF receptor agonists, whether they are modified antibodies or recombinant versions of their ligands has been shown to be essential for their activity [10]. As described above, members of the TNF ligand superfamily usually signal in a trimeric form that binds in a 1:1 stoichiometry to their respective TNF receptors, although higher order interactions are frequently required for their biological function, as was initially shown for the apoptosis inducing TNF superfamily Fas ligand (FasL) [42]. To obtain such multimeric forms of the ovine OX40L and CD70 molecules we used a previously employed strategy [43] in which the predicted extracellular TNF homology domain from human OX40L is fused to an isoleucine zipper trimerisation motif [32,44] to stabilise trimerisation of the THD followed by a human IgG1 derived Fc domain, which in our case was replaced with an Fc domain from the ovine species. In the case of the human recombinant protein, this was shown to adopt a functional hexameric structure formed by two OX40L trimers and three disulphide bonded Fc dimers. Stabilising isoleucine zipper trimerisation motifs have been tested before in other chimeric TNFLSF members such as the human CD40L [45] and TRAIL [46] proteins, generating higher biological activity molecules. This suggested that such a strategy would have a broad applicability to members of the family from other species, including the ovine CD70 tested in this report. While we did not assess the oligomeric nature of the purified *rOa*OX40L and *rOa*CD70 proteins, non-reducing SDS-PAGE indicated the presence of disulphide bonded oligomers as in the case above. This suggests that the ovine proteins may adopt also this hexameric structure, or at least a trimeric structure. The oligomeric state of *rOa*OX40L and *rOa*CD70 was competent to trigger signalling in cells expressing the cognate receptor. Additionally, these recombinant proteins may be used to monitor expression of OX40 and CD27 on the surface of ovine cells.

As a further step for the use of the secreted *rOa*OX40L and *rOa*CD70 as immunostimulators, we developed recombinant human adenovirus 5 based vectors that express them. Cell culture assays showed that infection with either recombinant virus produced secreted and bioactive proteins equivalent to the purified proteins described before. Human adenovirus 5 has been extensively used in vivo as a sustained gene delivery method in vivo [27,30,47]. While its major drawback for clinical use in humans is the widespread pre-existing immunity in the population [48,49,50], this is not the case for sheep, as this host is unlikely to have been exposed to the recombinant vector [30,51,52]. For example, inoculation of adenoviral vectors expressing bovine IFN type III has been shown to protect cattle from a subsequent FMDV challenge [53]. While the use of the recombinant cytokines interleukin 1 beta (IL1β) and TNFα as effective adjuvants in vaccination of ruminant species has already been proposed [54,55], their use has so far not been widely adopted. Possibly the use of specifically targeted costimulatory molecules such as the *Oa*OX40L and *Oa*CD70 constructs described here might provide improved vaccine enhancement in vivo. It is worth noting that a recombinant adenovirus expressing trimeric TNFSF ligands 4-1BBL or BAFF was found to effectively enhance anti Gag response in a murine HIV vaccine model [56], showing that adenoviral delivery of soluble TNF ligands can promote adjuvancy.

An important role of the OX40/OX40L costimulatory pathway is the generation of CD4^+^ T cell responses including memory cell generation [5]. OX40 is known to control the number of effector T cells that form during primary or secondary immune responses as well as the frequency of memory cells that are generated (reviewed in [4,6]). These reported activities of OX40 are relevant to vaccination protocols and activation of this ligand has therefore been proposed as a potent adjuvant strategy. A good example of OX40 role was demonstrated in vaccinia virus infection in mice, where OX40 was found to be critical for the development of CD8^+^ T cell responses against dominant and subdominant epitopes as well as for the generation of memory cells [57]. Relevantly, activation of OX40 signalling by an agonist antibody during vaccination with an attenuated poxviral vector provided enhanced effector and memory cell numbers and allowed complete protection against a lethal respiratory virus infection [58]. In this report, we show a consistent costimulatory activity on sheep PBMC, with activation of CD4^+^ and CD8^+^ cells by the newly developed ovine agonist.

A similar case can be made for the CD70-CD27 signalling axis that is known to enhance primary anti-viral CD8^+^ T cell responses and generation of memory T cells in mice and probably humans (reviewed in [11]). Recently, the generation of EBV specific T cell responses was shown to be dependent on this signalling pathway in a patient with EBV-driven recurrent lymphoproliferative disorder who had a CD70 deficiency [59] and stimulation of CD27 using agonist antibodies has been shown to enhance therapeutic vaccination in cancer immunotherapy [60]. In the ovine model used here, we were able to show expression of the CD27 molecule on both CD4^+^ and CD8^+^ T cells as well as on a subset of NK cells and a fraction of B-cells, which suggests that its role in the development of the immune response may be equivalent to that described in mice and humans. In support of this notion and as in the case of the ovine OX40, the purified ovine ligand was found to be able to induce IFNγ secretion from activated ovine CD4^+^ and CD8^+^ cells, confirming their nature as costimulatory molecules.

While CD70-CD27 signalling, like OX40-OX40 signalling, share overall costimulatory activities, differences in their activation dynamics as well as expression patterns on cellular subsets suggest a set of non-overlapping and non-redundant roles in immune response activation that warrant their individual and combined studies in the context of vaccination processes. In particular, future research will need to address sheep disease models where T cell response is thought to play a critical role in protection, as is the case of the Bluetongue virus [28] or peste des petits ruminants virus [61] infections.

## 5. Conclusions

In summary, we provided the first characterization of the ovine costimulatory OX40L-OX40 and CD70-CD27 signalling axes as well as generated reagents for their activation in vivo that may be useful for the enhancement of vaccination induced immune responses in sheep and other ruminant species.

## Supplementary Materials

The following are available online at https://www.mdpi.com/2076-393X/8/2/333/s1, Figure S1: A. Multiple sequence alignment of mammalian OX40R (TNFRSF4) proteins B. Multiple sequence alignment of mammalian CD27 (TNFRSF7) proteins C. Multiple sequence alignment of mammalian OX40L (TNFSF4) proteins D. Multiple sequence alignment of mammalian CD70 (TNFSF7) proteins.
