# Supplementary material for: Activation of OX40 and CD27 Costimulatory Signalling in Sheep through Recombinant Ovine Ligands
Source: Vaccines (Basel). 2020 Jun 22;8(2):333. doi: 10.3390/vaccines8020333 (PMC7350415; doi:10.3390/vaccines8020333)
Supplement: Supplementary file 1 [file vaccines-08-00333-s001.pdf]

Supplementary Figure 1

A. Multiple sequence alignment of mammalian OX40R (TNFRSF4) proteins

```
OX40R_HUMAN      MCVGARRLGRGPCAALLLLGLGLST-VTGLHCVGDTYPSNDRCCHECR-PGNGMVSRCSR   58
OX40R_MOUSE      MYVWVQQ-----PTALLLLALTLGVTARRLNCVKHTYPSGHKCCRECQ-PGHGMVSRCDH   54
OX40R_PIG        MRVGAQP-PRALSSALLLLGLVLGA-VARLTCVGDTYPSGRRCCKECQ-PGYGMERRCTD   57
OX40R_HORSE      MCVEAQR-PRAPCAALLLLGLVLGA-AAGQNCVGNTYPSGGRCCQECD-PGYGMESRCTQ   57
OX40R_BOVIN      MCVGTQP-PRAPGSALLLLGLVLGA-AAQPHCTGDTYPSGNRCCKECP-PGYGMESRCNH   57
OX40R_GOAT       MCVGTQP-PRAPGSALLLLGLVLSA-AAQPHCTGDTYPSGNRCCKECQ-PGYGMESRCIH   57
OX40R_SHEEP      MCVGTQP-PRAPGSALLLLGLVLSA-AAQPHCTGDTYPSGNRCCKECQPAGYGMESRCIH   58
                 * * .:       :*****.* *.. .    *. .****. :**:**   * **  **

OX40R_HUMAN      SQNTVCRPCGPGFYNDVVSSKPCKPCTWCNLRSGSERKQLCTATQDTVCRCRAGTQPLD-   117
OX40R_MOUSE      TRDTLCHPCETGFYNEAVNYDTCKQCTQCNHRSGSELKQNCTPTQDTVCRCRPGTQPRQD   114
OX40R_PIG        TQETECSRCKPGFYNEAVNYEPCKPCTQCNQRSGSEVKQRCTNTTDTVCGCRPGTQPQDG   117
OX40R_HORSE      RKDTVCLPCKPGYYNEATNYEACKPCTQCNQRSGSEPKQRCTSTQDTVCRCRPGTQPKH-   116
OX40R_BOVIN      NQDTVCSPCKPGYYNEAVNYEPCKPCTQCSQRSGSEPKQRCTPTRDTVCGCRPGSQPQDS   117
OX40R_GOAT       NRDTVCSPCKPGYYNEAVNYEPCKPCTQCSQRSGSEPKQRCTPTRDTVCGCRPGSQPQDS   117
OX40R_SHEEP      NRDTVCSPCKPGYYNEAVNYEPCKPCTQCSQRSGSEPKQRCTPTRDTVCGCQAGQASATN   118
                  ::* *  *  *:**:... . ** ** *. ***** ** ** * **** *: *

OX40R_HUMAN      -SYKPGVDCAPCPPGHFSPGDNQACKPWTNCTLAG-KHTLQPASNSSDAICEDRDPPATQ   175
OX40R_MOUSE      SGYKLGVDCVPCPPGHFSPGNNQACKPWTNCTLSG-KQTRHPASDSLDAVCEDRSLLATL   173
OX40R_PIG        FGYKRGVDCVRCPPGHFSPGNDQACQPWTNCTLVG-KRTLQAASNSSDAVCEDRSPPATP   176
OX40R_HORSE      -GYKLGVDCAPCPPGHFSPGNNQACKPWTNCTSVG-KRTLQLASNSSDAVCEDRSPPATP   174
OX40R_BOVIN      YGYKRGVDCAPCPPGHFSPGNDQACQPWTNCTLLG-KRTLRAANSSSDAICEDRSPPATP   176
OX40R_GOAT       YGYKRGVDCTPCPPGHFSPGNDQACQPWTNCTLLG-KRTLRAANSSSDAICEDRSPPATP   176
OX40R_SHEEP      VELVRGPYCAPCPPGHFSPGNDQACQPWTNPCPTPLCDGPCPHHNSSDAICEDRSPPATP   178
                      *  *. *********::***:****              .* **:****.  **

OX40R_HUMAN      PQETQGPPARPITVQPTEAWPRTSQGPSTRPVEVPGGRAVAAILGLGLVLGLLGPLAILL   235
OX40R_MOUSE      LWETQRPTFRPTTVQSTTVWPRTSELPSPPTLVTPEGPAFAVLLGL--GLGLLAPLTVLL   231
OX40R_PIG        PQETQGPPARSPTARPTTAWPRTSQEPSTPHTESPKGPELAAVLGLGLGLVLLAPVAAVL   236
OX40R_HORSE      PWETQGPPAWAPTTQPTTSWPRVSQGPSTPPTEPPRGPELAAILGLALGLGLLAPVAAVL   234
OX40R_BOVIN      PWETQGPPVQSTTAKPTTSWSKASQGSSMPHTEPPKAPELSAVLGLGLGLGLLAPVAAML   236
OX40R_GOAT       PWETQGPPVQSTTAKPTTSWSKASQGSSMPHTEPPKAPELSAVLGLGLGLGLLAPVAAML   236
OX40R_SHEEP      PWETQGPPVQSTTAKPTTSWSKASQGSSMPHTEPPKAPELSAVLGLGLGLGLLAPVAAML   238
                   *** *     *.: *  * :.*:  *      * .  .:.:***   * **.*:: :*

OX40R_HUMAN      ALYLLRRDQRLPPDAHKPPGGGSFRTPIQEEQADAHSTLAKI 277
OX40R_MOUSE      ALYLLRKAWRL-PNTPKPCWGNSFRTPIQEEHTDAHFTLAKI 272
OX40R_PIG        VLLLHHRAWRLLPNAPKPPRENGFRIPIQEEHADANSSLAKT 278
OX40R_HORSE      ALFLHHRAWRLPPNVPKPPGGNSFRTPIQEEHADANSTLAKI 276
OX40R_BOVIN      ALLLHHRAWRLLTNTPKPPGGNSFRTPIQEEHTDANSSLAKI 278
OX40R_GOAT       ALLLHHRAWRLLTNTPKPPGGNSFRTPIQEEHTDANSSLAKI 278
OX40R_SHEEP      ALLLHHRAWRLLTNTPKPPGGNSFRTPIQEEHTDANSSLAKI 280
                 .* * ::  **  :. **   ..** *****::**: :***
```

Sequences were retrieved from Uniprot (https://www.uniprot.org) with the accession numbers in brackets as follows: OX40R_HUMAN (P43489), OX40R_MOUSE (P47741|TNR4_MOUSE), OX40R_PIG (A0A287A7B3), OX40R_HORSE (A0A5F5PWA8), OX40R BOVIN (A5PJH6), OX40R_GOAT (A0A452FBG5) and OX40R_SHEEP (W5P810). Multiple sequence alignments were performed using Clustal omega software (https://www.ebi.ac.uk/Tools/msa/clustalo/) under default settings. Fully conserved sites are indicated with asterisks. The following elements have been highlihghted on the sequence of the human orthologue: transmembrane domain in red letters, full TNFR repeats as identified by SMART (SM000208) by blue lines above the sequence and a TRAF binding motif in blue letters.

B. Multiple sequence alignment of mammalian CD27 (TNFRSF7) proteins

```
CD27_HUMAN      MARPHPWWLCVLGTLVGLSATPAPKSCPERHYWAQGKLCCQMCEPGTFLVKDCDQHRKAA    60
CD27_MOUSE      MAWPPPYWLCMLGTLVGLSATLAPNSCPDKHYWTGGGLCCRMCEPGTFFVKDCEQDRTAA    60
CD27_PIG        MARSPLCWLWVLGTLAGLSATPAPQSCPEKHYWARGELCCQMCQPGTFLVKDCDQHGKAA    60
CD27_HORSE      MARPPPCWLWVLGTLAGLSATPAPKSCPEKHYWARGELCCPMCKPGMFLKEDCDGHGRIT    60
CD27_BOVIN      MARLPPCWLWVLGTLAGLSATPDPKSCPEKHYWAQGGWCCQMCEPGTFLVKDCEQHREAA    60
CD27_GOAT       MARLPPCWLWVLGTLAGLSATPGPKSCPEKHYWAQGGWCCQMCEPGTFLVKDCEQHGEAA    60
CD27_SHEEP      MAWLPPCWLWVLGTLAGLSATPGPKSCLEKHYWAQGGWCCQMCEPGTFLVKDCEQHGEAA    60
                **     ** :****.*****  *:** ::***: *  ** **:** *: :**: .   :

CD27_HUMAN      QCDPCIPGVSFSPDHHTRPHCESCRHCNSGLLVRNCTITANAECACRNGWQCRDKECTEC    120
CD27_MOUSE      QCDPCIPGTSFSPDYHTRPHCESCRHCNSGFLIRNCTVTANAECSCSKNWQCRDQECTEC    120
CD27_PIG        RCDPCKQGVSFSPDYHSRPHCESCRHCNSGLFVQNCTLTANAKCACPEGWQCRDKECTEC    120
CD27_HORSE      QCDSCIPGVSFSPDYHARPHCESCRHCNSGLLIRNCTLTANTECGCPKGWQCKDKECTEC    120
CD27_BOVIN      QCNPCTPGVSFMPDHHSRPHCESCRHCNSGLLIRNCTLTANSECACPEGQQCRDKDCMEC    120
CD27_GOAT       QCDPCTPGVSFTPDHHSRPHCESCRHCNSGLLIRNCTLTANSECACPEGQQCRDKDCMEC    120
CD27_SHEEP      QCDPCTPGVSFTPDHHSRPHCESCRHCNSGLLIRNCTLTANSKCACPEGQQCRDKDCMEC    120
                :*: *  *.** **:*:*************::::***:***::*.* :. **:*::* **

CD27_HUMAN      DPLPNPSLTARSSQALSPHPQPTHLPYVSEMLEARTAGHMQTLADFRQLPARTLSTHWPP    180
CD27_MOUSE      DPPLNPALTRQPSETPSPQPPPTHLPHGTEKPSW---------PLHRQLPNSTVYSQRSS    171
CD27_PIG        DGPA---------QTPGPHPQPSHSPYAEAIPEARTSRYTQTLADVGQVPVPTISTYLSS    171
CD27_HORSE      DPPANPSLTPRPSQA--PGPQPTHLPYAKKMPEARTVRHVQTLADFRQLPAPVLSTHWPP    178
CD27_BOVIN      DGPA---------QAPGPHPQPSHLPYAEEIAESRTDRHTQTLANSRWLPAPTLSTHWSP    171
CD27_GOAT       DGPA---------QAPGPHPQPSQLPYAEEIPEARTDRHTQTLANSRWLPPATLSTHWSP    171
CD27_SHEEP      DGPA---------QAPGPHPQPSQLPYAEEIPEARTDRHTQTLANSRWLPPATLSTHWSP    171
                *            ::  * * *:: *:     .               :*  .: :

CD27_HUMAN      QRSLCSSDFIRILVIFSGMFLVFTLAGALFLHQRRKYRSNKGESPVEPAEPCHYSCPREE    240
CD27_MOUSE      HRPLCSSDCIRIFVTFSSMFLIFVLGAILFFHQRRNHGPNEDRQA-VPEEPCPYSCPREE    230
CD27_PIG        Q--RCSSECIRILVVLSGVFLAFTVVGALFLYQQRKYGLNTGECPVAPAEPCPYSYPREE    229
CD27_HORSE      QRSLCSTDCIRIFVIFSGMFLAFTMVGALFLHQQRKYGLNKGESPA-VAEPGPYSCPREE    237
CD27_BOVIN      QRSLCSANCIRIFVLLSGMFLAFTIVGALFLHQQRK--LNAGESPVAPAEPCPYTCPSEE    229
CD27_GOAT       QRSLCSVNCVRIFVLLSGMFLAFTIVGALFLHQQRK--LNAGESPVAPAEPCPYTCPSEE    229
CD27_SHEEP      QRSLCSVNCVRIFVLLSGMFLAFTIVGALFLHQQRK--LNAGESPVAPAEPCPYTCPSEE    229
                :   ** : :**:* :*.:** *.: . **::*:*:   * ..      **  *: * **

CD27_HUMAN      EGSTIPIQEDYRKPEPACSP---------------------   260
CD27_MOUSE      EGSAIPIQEDYRKPEPAFYP---------------------   250
CD27_PIG        EGSAIPIQEDYRKPELASYL---------------------   249
CD27_HORSE      EGSAFPIQEDYRKPEPASYP---------------------   257
CD27_BOVIN      EGSAIPIQEDYRKPEPTSYF---------------------   249
CD27_GOAT       EGSAIPIQEDYRKPELTSYSEPVLLREGHHCNQVLASTSLH   270
CD27_SHEEP      EGSAIPIQEDYRKPELTSYSEPVLLREGHHCNQVLASTSPH   270
                ***::********** :
```

Sequences were retrieved from Uniprot (https://www.uniprot.org) with the accession numbers in brackets as follows: CD27_HUMAN (P26842), CD27_MOUSE (P41272), CD27_PIG (F1SL30), CD27_HORSE (F7BWA6), CD27_BOVINE (F1N4E2), CD27_GOAT (A0A452FJN6) or from Genbank (https://www.ncbi.nlm.nih.gov/) for CD27_SHEEP (XP_004006990.1). Multiple sequence alignments were performed using Clustal omega software (https://www.ebi.ac.uk/Tools/msa/clustalo/) under default settings. Fully conserved sites are indicated with asterisks. The following elements have been highlihghted on the sequence of the human orthologue: transmembrane domain in red letters, full TNFR repeats as identified by SMART (SM000208) by blue lines above the sequence and a TRAF binding motif in blue letters.

C. Multiple sequence alignment of mammalian OX40L (TNFSF4) proteins

```
OX40L_HUMAN      --MERVQPLEENVGNAARPRFERNK-LLLVASVIQGLGLLLCFTYICLHFSALQVSHRYP   57
OX40L_MOUSE      MEGEGVQPLDENLENGSRPRFKWKKTLRLVVSGIKGAGMLLCFIYVCLQLSS--SPAKDP   58
OX40L_PIG        --MEGVQPLDENVGNAPGRRLLRNK-LLLVASVIQGLGLLLCLTYICLHLYA-QVPSQYP   56
OX40L_HORSE      --MEGVQPLEENVGNTPGRRFQRNK-LLLVTSIIQGLGLLLCLTYVCLHFYTSQVPSQYP   57
OX40L_BOVIN      MKMEGVQPLDENVGNVPGRRFLRNK-LLLVASIIQGLGLLLCLTYICLHFYA-QVPSQYP   58
OX40L_GOAT       --MEGVQPLDENVGNAPGRRFLRNK-LLLVASIIQGLGLLLCLTYICLHFYA-QVPSQYP   56
OX40L_SHEEP      --MEGVQPLDENVGNAPGRRFLRNK-LLLVASIIQGLGLLLCLTYICLHFYA-QVPSQYP   56

                    * ****:**: *    *:  :* * **.* *:* *:***: *:**:: :     : *

OX40L_HUMAN      RIQSIKVQFTEYKKEKGFILTSQKEDEIMKVQNNSVIINCDGFYLISLKGYFSQEVNISL   117
OX40L_MOUSE      PIQRLRGAVTRCEDGQLFISSYKNEYQTMEVQNNSVVIKCDGLYIIYLKGSFFQEVKIDL   118
OX40L_PIG        PIQSIKVQFTKCENDNGFIITPSSKDGTMKVQNNSIIINCDGFYLISLKGYFSQELSLML   116
OX40L_HORSE      PIQSIRVQFTSCENEKGFIITSPNQDEIMKVQDNSIIINCDGFYLISLKGYFSQELSLSL   117
OX40L_BOVIN      PIQSIRVQFTKCENENGFIITSPDADGTMKVQNNSIIITCDGFYLISLKGYFSQELSLRL   118
OX40L_GOAT       PIQSIRVRFTKCENENGFIITSPDADGTMKVQNNSIIITCDGFYLISLKGYFSQKLSLRL   116
OX40L_SHEEP      PIQSIRVRFTKCENENGFIITSPDADGTMKVQNNSIIITCDGFYLISLKGYFSQKLSLRL   116

                  ** ::  .*  :. : ** :  .    *:**:**::*.***:*:* *** * *::.: *

OX40L_HUMAN      HYQKDEEPLFQ--LKKVRSVNSLMVASLTYKDKVYLNVTTDNTSLDDFHVNGGELILIHQ   175
OX40L_MOUSE      HFREDHNPISIPMLNDGRRIVFTVVASLAFKDKVYLTVNAPDTLCEHLQINDGELIVVQL   178
OX40L_PIG        QYRKGRKPLFS--LNKVKSVDSVTVADLAFKDKVFLNVTTHSASCEDIQVNGGELILIHQ   174
OX40L_HORSE      HYRKGREPLSS--LSKVRSVNSIMVAYLAFKDKVYLNVTTHNTSCDDIQVNGGELILIHQ   175
OX40L_BOVIN      LYRKGREPLFS--LNMVKIVDSVTVAYLRFKDKVYLNMTTQNASCEDIQVNGGELILIHQ   176
OX40L_GOAT       LYRKGREPLFS--LNMVKIVDSVTVAYLRFKDKVYLNVTTQNASCEDIQVNGGELILIHQ   174
OX40L_SHEEP      LYRKGREPLFS--LNMVKIVDSVTVAYLRFKDKVYLNVTTQNASCEDIQVNGGELILIHQ   174

                 :::..:*:    *.  : :    ** * :****:*.:.: .:  :.:::*.****:::

OX40L_HUMAN      NPGEFCVL-------------  183
OX40L_MOUSE      TPG-YCAPEGSYHSTVNQVPL  198
OX40L_PIG        NPGGFCVY-------------  182
OX40L_HORSE      NPGGFCAY-------------  183
OX40L_BOVIN      NPGGFCVY-------------  184
OX40L_GOAT       NPGGFCVY-------------  182
OX40L_SHEEP      NPGGFCVY-------------  182

                 .** :*.
```

Sequences were retrieved from Uniprot (https://www.uniprot.org) with the accession numbers in brackets as follows: OX40L_HUMAN (P23510), OX40L_MOUSE (P43488), OX40L_PIG (Q4QTJ8), OX40L_HORSE (F6UJR4), OX40L__BOVIN (E1BGY4), OX40L_GOAT (A0A452FAW5) and OX40L_SHEEP (W5PZ67). Multiple sequence alignments were performed using Clustal omega software (https://www.ebi.ac.uk/Tools/msa/clustalo/) under default settings. Fully conserved sites are indicated with asterisks. For human and ovine sequences, transmembrane domains are indicated in red letters and TNF homology domains (SMART TNF domain SM000207) in green letters. The predicted extracellular region of the ovine orthologue that was cloned is indicated by a blue line under the text.

D. Multiple sequence alignment of mammalian CD70 (TNFSF7) proteins

```
CD70_HUMAN      ------------------------------------------------------------    0
CD70_MOUSE      ------------------------------------------------------------    0
CD70_PIG        ------------------------------------------------------------    0
CD70_HORSE      ---------------------------------------------------------MQR    3
CD70_BOVIN      MVERAWSLPLTDIPREGHGWSLAGSDRLRGPRGCEKAEASFPLPACPLRTLPAATQLMKC    60
CD70_GOAT       MVERAWSLPLTDIPREEHGWSLAGSDRPREPCGCEKAEASFPLPACPLRTLPATTQLMKC    60
CD70_SHEEP      ---------------------------------------------------------MKC    3


CD70_HUMAN      ------MPEEGSGCSVRRRPYGCVLRA---ALVPLVAGLVICLVVCIQRFAQAQQQLPLE    51
CD70_MOUSE      ------MPEEGRPCPWVRWSGTAFQRQWPWLLLVVFITVFCCWFHCSGLLSKQQ-QRLLE    53
CD70_PIG        ------MEEEGSGCNVPRLPWASILRA---ALLLLLIGMVIYCFLCGQRFTQ-Q---QLD    47
CD70_HORSE      RPR-TAMAEEGSGCLVRRLPWVTILRV---AFLLILIGMVIYCFVCNQRLAQQQ---QLE    56
CD70_BOVIN      SWRTMMAPEEAASCQVPRWPWASILRV---TISVLLSTG-TCYLVCNLCFSQQQ---QLD    113
CD70_GOAT       SWKTVMATEEAASCQVTRRPWASILRV---TIPVLLSIG-TCYLICTLCFRQQQ---QLD    113
CD70_SHEEP      SWRTIMATEEAASYQVNRWPWASILRV---IIPVLLSIG-TCYLVCNLCFRQQQ---QLD    56

                        **.      *     . *     :  :.       . *   : : *    *:

CD70_HUMAN      SLGWDVAELQLNHTGPQQDPRLYWQGGPALGRSFLHGPELD-KGQLRIHRDGIYMVHIQV    110
CD70_MOUSE      HPEPHTAELQLNLTVPRKDPTLRWGAGPALGRSFTHGPELE-EGHLRIHQDGLYRLHIQV    112
CD70_PIG        STGWDLAELLLNHTESRQDPRLRWQGSPALGRSFVHGPELD-NGQLRIQRTGIYRLHIQV    106
CD70_HORSE      SSGWAVAELQLNHTGPRHDARLHWQGNPALGRSFVHGLELD-YGQLRIQHPGIYRLHIQV    115
CD70_BOVIN      STRWDLAELQLNHTGSRQDPRLPWQGSPALGRSFLHGPKLDDNGQLQIQRDGIYRLHIQV    173
CD70_GOAT       STRWDLAELQLNHTGSRQDPRLRWQGSPALGRSFLHGPELDDNGQLRIQRDGIYRLHIQV    173
CD70_SHEEP      STRWDLAELQLNHTGSRQDPRLRWQGSPALGRSFLHGPELDDNGQLRIQRDGIYRLHIQV    116

                      *** ** *  ::*  * * ..******* ** :*:  *:*:*:: *:* :****

CD70_HUMAN      TLAICSSTTA-SRHHPTTLAVGICSPASRSISLLRLSFHQ-GCTIASQRLTPLARGDTLC    168
CD70_MOUSE      TLANCSSPGS-TLQHRATLAVGICSPAAHGISLLRGRFGQ-DCTVALQRLTYLVHGDVLC    170
CD70_PIG        TLTNCSSSTWTVMPRQATLTLGICSPTTHSISLLRLNFHH-TCRVASQRLTPLAKGDVLC    165
CD70_HORSE      TLTNCS-STWTNAVRRATLAVGICSSATHSISLLRLNFYN-ACTVASQRLTYLAKGDTLC    173
CD70_BOVIN      TLANCSSSTWTAEPQRATLTVAICSPAAHSISLLRLSFHRGGCWVASQRLTFLARGDILC    233
CD70_GOAT       TLANCSSSTWTAEPQRATLTVAICSPAAHSISLLRLSFHRGACWVASQRLTFLALGDILC    233
CD70_SHEEP      TLANCSSSTWTAEPQRATLTVAICSPAAHSISLLRLSFHRGACWVASQRLTFLAHGDILC    176

                **: **        : :**::.*** :::.*****  * .  * :* **** *. ** **

CD70_HUMAN      TNLTGTLLPSRNTDETFFGVQWVRP    193
CD70_MOUSE      TNLTLPLLPSRNADETFFGVQWICP    195
CD70_PIG        TNLTLPLLPSRNADETFFGVQLVRP    190
CD70_HORSE      TNLTLPWLPSRNSDETFFGVQWVHL    198
CD70_BOVIN      TNLTLPLLPSRNADETFFGIQWVHP    258
CD70_GOAT       TNLTLPLLPSRNADETFFGIQWVHP    258
CD70_SHEEP      TNLTLPLLPSRNTDETFFGIQWVRP    201

                ****   *****:******:* :
```

Sequences were retrieved from Uniprot (https://www.uniprot.org) with the accession numbers in brackets as follows: CD70_HUMAN (P32970), CD70_MOUSE (O55237), CD70_PIG (Q3ZDR4), CD70_HORSE (F6XHL7), CD70_BOVIN (E1B972), CD70_GOAT (A0A452G8J0) and CD70_SHEEP (W5P639). Multiple sequence alignments were performed using Clustal omega software (https://www.ebi.ac.uk/Tools/msa/clustalo/) under default settings. Fully conserved sites are indicated with asterisks. For human and ovine sequences, transmembrane domains are indicated in red letters and TNF homology domains (SMART TNF domain SM000207) in green letters. The predicted extracellular region of the ovine orthologue that was cloned is indicated by a blue line under the text.
